# Self-Perceptions of Driving Ability in Older Adults With and Without Alzheimer’s Disease

**DOI:** 10.1093/geroni/igaa057.536

**Published:** 2020-12-16

**Authors:** Rebecca Davis, Megan Owens

**Affiliations:** Grand Valley State University, Grand Rapids, Michigan, United States

## Abstract

A concern for families and persons with dementia is determining when driving cessation is necessary. Older adults often modify driving behaviors as they age due to self-perception of a decline in their abilities. However, it is not known how persons with Alzheimer’s disease modify driving behaviors. The purpose of this study was to determine difference in how similarly aged persons with and without Alzheimer’s disease perceive their driving behaviors and modify them. Data were collected for 48 persons with early stage AD or mild cognitive impairment (MCI) due to AD; and 53 similarly aged older adults without cognitive diagnoses, using a survey on driving frequency, habits, and wayfinding ability. Chi-square analysis was used to determine any statistically significant differences in reported driving habits and difficulties between the study groups. Results from the analysis showed that the majority of the sample was still driving (n=81, 83%). Persons with AD/MCI (n=33, 73%) were less likely to be driving than the control group (n=48, 91%). Of those who were still driving, those with AD/MCI were more likely to limit driving frequency, avoid driving alone and in unfamiliar routes. Worries about getting lost were more frequently noted in those with AD/MCI. The AD group was more likely to make modifications to their driving such as limiting driving to daylight hours and not driving alone. These important results indicate persons with early stage AD/MCI may have self-awareness about driving impairments and make modifications to their driving habits that may increase their driving safety.

